# Inference of Cross-Species Gene Flow Using Genomic Data Depends on the Methods: Case Study of Gene Flow in *Drosophila*

**DOI:** 10.1093/sysbio/syaf019

**Published:** 2025-05-27

**Authors:** Jiayi Ji, Thomas Roberts, Tomáš Flouri, Ziheng Yang

**Affiliations:** Department of Genetics, Evolution, and Environment, University College London, Gower Street, London WC1E 6BT, UK; Department of Genetics, Evolution, and Environment, University College London, Gower Street, London WC1E 6BT, UK; Department of Genetics, Evolution, and Environment, University College London, Gower Street, London WC1E 6BT, UK; Department of Genetics, Evolution, and Environment, University College London, Gower Street, London WC1E 6BT, UK

**Keywords:** bpp, *Drosophila*, introgression, migration, multispecies coalescent, MSC-I, MSC-M

## Abstract

Analysis of genomic data in the past two decades has highlighted the prevalence of introgression as an important evolutionary force in both plants and animals. The genus *Drosophila* has received much attention recently, with an analysis of genomic sequence data revealing widespread introgression across the species phylogeny for the genus. However, the methods used in the study are based on data summaries for species triplets and are unable to infer gene flow between sister lineages or to identify the direction of gene flow. Hence, we reanalyze a subset of the data using the Bayesian program bpp, which is a full-likelihood implementation of the multispecies coalescent model and can provide more powerful inference of gene flow between species, including its direction, timing, and strength. While our analysis supports the presence of gene flow in the species group, the results differ from the previous study: we infer gene flow between sister lineages undetected previously whereas most gene-flow events inferred in the previous study are rejected in our tests. To verify our conclusions, we performed simulations to examine the properties of Bayesian and summary methods. Bpp was found to have high power to detect gene flow, high accuracy in estimated rates of gene flow, and robustness under misspecification of the mode of gene flow. In contrast, summary methods had low power and produced biased estimates of introgression probability. Our results highlight an urgent need for improving the statistical properties of summary methods and the computational efficiency of likelihood methods for inferring gene flow using genomic sequence data.

Introgression—cross-species gene flow via hybridization and backcrossing—challenges the classical view of species as reproductively isolated entities. Historically, zoologists disregarded its importance, arguing that “introgression is rare and probably negligible as an evolutionary factor” (Mayr, 1963). Underlying this perception is the observation that hybrids are often far less fertile than the parental species. However, recent studies analyzing genomic sequence data have found hybridization or introgression to be pervasive across the tree of life, from Neotropical orchids (Pinheiro *et al.*, 2010), to mosquitoes (Fontaine *et al.*, 2015; Thawornwattana *et al.*, 2018), Cichlid fishes (Malinsky *et al.*, 2018), *Panthera* cats (Figueiro *et al.*, 2017), and ancient Hominins (Green *et al.*, 2010).

Gene flow, in addition to ancestral polymorphism, may cause gene trees to differ from the species tree (Leaché *et al.*, 2014; Long and Kubatko, 2018; Jiao *et al.*, 2020), posing challenges to species tree inference. The need to infer species phylogenies despite genealogical discordance across the genome, along with a desire to unravel the role of introgression in the history of species divergence and ecological adaptation, has motivated the development of statistical methods to detect gene flow and to estimate its rate (see Jiao *et al.*  2021; Hibbins and Hahn  2022 for reviews).

Summary methods for inferring gene flow and estimating its strength use summaries of genomic data, such as frequencies of gene trees or average genome-wide distances between populations. Most summary methods operate on species triplets (or quartets if an outgroup is included). For example, the D-statistic (or ABBA-BABA test) (Green *et al.*, 2010) and HyDe (Blischak *et al.*, 2018) test for gene flow using genome-wide site-pattern counts in a species quartet. HyDe assumes a hybrid-speciation model with symmetrical population sizes (Blischak *et al.*, 2018; Ji *et al.*, 2023). SNaQ (Solis-Lemus and Ane, 2016; Solis-Lemus *et al.*, 2017) is a pseudo-likelihood method that uses reconstructed gene tree topologies (see also Yu *et al.*, 2012), while QuIBL (Edelman *et al.*, 2019) uses estimated internal branch lengths in gene trees. Summary methods are computationally efficient but have two limitations. First, they use only a portion of the information in the multilocus sequence data. For example, D and HyDe use a few site-pattern counts pooled across the genome but ignore information concerning gene flow in the variation of genealogical history across the genome (Lohse and Frantz, 2014; Shi and Yang, 2018; Zhu and Yang, 2021). SNaQ uses gene-tree topologies and ignores information in gene-tree branch lengths or coalescent times. Second, summary methods based on gene trees (e.g., SNaQ and QuIBL) do not accommodate the uncertainties and errors in reconstructed gene trees, which may be considerable when the species are closely related and the sequences are highly similar. Methods that use gene-tree branch lengths are in particular prone to random sampling errors (DeGiorgio and Degnan, 2014). Under the multispecies coalescent (MSC) model, gene trees are unobserved latent variables; one should average over them when inferring the species tree or estimating the rate of gene flow rather than estimating them using phylogenetic programs and treating the estimates as observed data. As a result of limitations such as those, most summary methods are unable to identify gene flow between sister lineages, or to infer the direction or timing of gene flow (Degnan, 2018; Jiao *et al.*, 2021; Yang and Flouri, 2022; Ji *et al.*, 2023; Pang and Zhang, 2024). Summary methods may be useful to suggest candidate introgression scenarios but may have limitations when used to characterize the complex history of species divergence and between-species gene flow when data are available from many species.

In contrast, likelihood methods under the MSC make a full use of information in multilocus sequence alignments (Degnan, 2018; Rannala *et al.*, 2020; Jiao *et al.*, 2021). Both the MSC-introgression (MSC-I) and the MSC-migration (MSC-M) models are implemented in the Bayesian Markov chain Monte Carlo (MCMC) program bpp (Flouri *et al.*, 2020, 2023). The MSC-I model assumes that gene flow occurs as a major discrete event at a certain time point (Wen and Nakhleh, 2018; Zhang *et al.*, 2018; Flouri *et al.*, 2020). The MSC-M model assumes continuous gene flow that occurs over extended time periods (Hey *et al.*, 2018; Flouri *et al.*, 2023). In simulations, bpp produced accurate estimates of introgression probabilities and introgression times (Huang *et al.*, 2020, 2022a) and showed high power for detecting gene flow (Ji *et al.*, 2023; Pang and Zhang, 2024). While likelihood methods involve intense computation, recent implementations of computationally efficient algorithms in bpp have made it possible to analyze large datasets with thousands of loci. The current version of bpp has the limitation that a full parametric model of gene flow must be specified, including the species tree, the introgression/migration events, and the species/populations involved. Programs such as PhyloNet (Wen and Nakhleh, 2018) and beast (Zhang *et al.*, 2018) have included MCMC moves between introgression models but are not feasible computationally except for very small datasets with <100 loci.

A recent phylogenomic analysis of protein-coding genes from *Drosophila* revealed widespread introgression across a phylogeny of 149 species (Suvorov *et al.*, 2022). The data were split into nine well-supported clades to detect gene flow within each. Several tests based on rooted triplets (or unrooted quartets) were employed, including two newly developed approaches: the discordant count test (DCT) and branch length test (BLT) (Suvorov *et al.*, 2022). Applied to species triplets, DCT appears to be equivalent to SNaQ (Solis-Lemus and Ane, 2016; Solis-Lemus *et al.*, 2017), while BLT is similar to QuIBL as both use estimated branch lengths in triplet gene trees. Another method used by Suvorov *et al.* (2022) is PhyloNet (Wen *et al.*, 2018), which takes inferred gene-tree topologies as input data and ignores information in coalescent times. Those methods cannot identify gene flow between sister lineages and cannot identify the direction of gene flow. As gene flow involving ancestral species may show up in many triplet tests, a heuristic metric called f-branch was used to move introgression events to ancestral branches in the given species tree (Malinsky *et al.*, 2018). The approach does not consider species divergence times or introgression times and may assign gene flow to donor and recipient populations that were not contemporary. Such limitations of the analytical methods used by Suvorov *et al.* (2022) suggest a need for reanalysis of the data using likelihood methods such as bpp. In a recent analysis of exonic data from six Rocky Mountain chipmunk species in the *Tamias* group, the summary method HyDe failed to detect any signal of gene flow affecting the nuclear genome, in contrast to the mitochondrial genome, which is well-known to be involved in rampant gene flow in the group, prompting discussions of cytonuclear discordance (Sarver *et al.*, 2021). However, a reanalysis of the same data using bpp detected robust evidence for multiple ancient introgression events affecting the nuclear genome, including one between sister species (Ji *et al.*, 2023), suggesting no evidence for cytonuclear discordance. Thus, analyses of the same data using summary (Sarver *et al.*, 2021) and Bayesian (Ji *et al.*, 2023) methods produced opposing biological conclusions. It is unclear whether the conclusions of Suvorov *et al.* (2022) are similarly affected by the use of summary methods.

Here, we apply the MSC-I and MSC-M models implemented in bpp (Flouri *et al.*, 2020, 2023) to reanalyze a subset of the *Drosophila* data of Suvorov *et al.* (2022). We used data from clade 2, which showed the strongest signal of introgression in the analysis of Suvorov *et al.* (2022, Table 1). Consistent with Suvorov *et al.* (2022), we detected strong evidence for gene flow, but the details differ. The strongest signature of introgression in our analysis is between two sister lineages, not detected by Suvorov *et al.* (2022), while several gene-flow scenarios inferred by Suvorov *et al.* (2022) are rejected in our test. To understand the differences in the results from the two studies, we conduct computer simulations to evaluate the statistical properties of bpp and the summary methods used by Suvorov *et al.* (2022), including HyDe, QuIBL, DCT, BLT, and SNaQ. Our results suggest that the different results may be explained by the lack of power of the summary methods used. Our study highlights the need and importance of using powerful statistical methods to infer gene flow using genomic datasets.

## Materials and Methods

### The *Drosophila* Dataset

**Fig 1:**
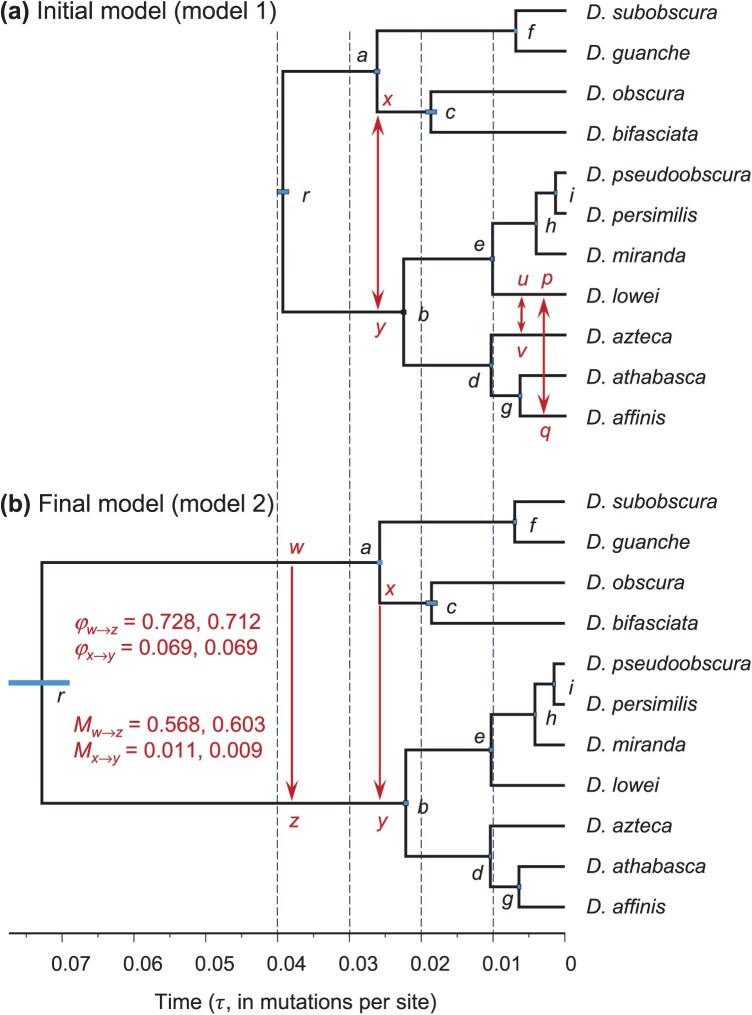
a) Species phylogeny for 11 *Drosophila* species in clade 2 of Suvorov *et al.* (2022) showing potential gene-flow events in our initial model. Arrows represent potential gene-flow events, based on analyses of species triplets by Suvorov *et al.* (2022) (Table S1) and on bpp estimates of divergence times. b) Final model of gene flow from our analysis, with two gene-flow events from branches ra to rb (i.e., w→z) and from ac to rb (x→y). Estimates of the introgression probability (φ) in the MSC-I model and migration rate (M) in the MSC-M model are from the two data halves. Branch lengths are proportional to posterior means of species divergence times and introgression times (τ, measured in mutations per site) with node bars representing the 95% HPD CIs, from bpp analyses of the first half of the data under the MSC-I model. Estimates for the second half are very similar. Estimates of all parameters under both the MSC-I and MSC-M models for the two data halves are in Table S4.

Suvorov *et al.* (2022) generated and compiled sequence alignments for 2794 single-copy protein-coding genes (BUSCO, for Benchmarking Universal Single-Copy Orthologs) from 155 *Drosophila* species and constructed a species phylogeny. Data for nine well-established clades were then used to infer interspecific gene flow. Here, we used data for clade 2 in the species tree, comprised of 11 species: *Drosophila affinis, Drosophila athabasca, Drosophila azteca, Drosophila lowei, Drosophila miranda, Drosophila persimilis, Drosophila pseudoobscura, Drosophila bifasciata, Drosophila obscura,*  *Drosophila guanche*, and *Drosophila subobscura* (Fig. 1a). Seventeen loci had <2 species and were removed, leaving 2777 loci. The 2777 loci were split into two random halves, with 1389 and 1388 loci, respectively, and analyzed separately.

### Inferring the *Drosophila* Species Phylogeny

We inferred the species tree under the MSC model with no gene flow using bpp (Yang and Rannala, 2014; Rannala and Yang, 2017). This is the A01 analysis of Yang (2015). The two data halves were analyzed separately. There are two types of parameters in the MSC model: species divergence times (τ) and population sizes (θ), both measured in the expected number of mutations per site. We assigned the gamma prior to the age of the species-tree root, $\tau_{R}∼$ G(2,50), with mean 2∕50=0.04. Given the age of the root, the other divergence times had the uniform-Dirichlet prior distribution (Yang and Rannala, 2010, eq. 2). A gamma prior is assigned to population size parameters on the species tree, θ∼ G(2,200), with mean 0.01. The JC mutation model (Jukes and Cantor, 1969) was used in the calculation of the likelihood for the sequence alignment at each locus. We expect JC to be sufficient for correcting for multiple hits at the same site because sequences from closely related species are highly similar (Shi and Yang, 2018; Flouri *et al.*, 2022) (see below for further tests). We used a burn-in of 40,000 MCMC iterations and then took $2\times 10^{5}$ samples, sampling every 2 iterations. Each analysis was repeated four times, with convergence of the MCMC confirmed by consistency across runs.

### Constructing a Model of Gene Flow for the *Drosophila* Data

Species tree inference using bpp produced a well-supported species phylogeny, which had the same topology as inferred by Suvorov *et al.* (2022) (Figs 1a and S1). The species phylogeny appeared to be unaffected by gene flow. We thus added candidate gene-flow events onto this binary species tree, using a procedure similar to that followed by Ji *et al.* (2023) in their analysis of a chipmunk genomic dataset. We assessed the gene-flow scenarios proposed by Suvorov *et al.* (2022, Fig. 3) by integrating their DCT/BLT analyses of many species triplets (Table S1), with reference to estimated species divergence times from bpp. The triplet methods of Suvorov *et al.* (2022) are unable to identify the direction of gene flow (e.g., Thawornwattana *et al.*, 2023; Pang and Zhang, 2024). Thus, we assumed bidirectional gene flow in our initial model, with the expectation that if the gene-flow event in a particular direction is nonexistent, the estimated rate of gene flow will be close to zero and the Bayesian test will reject gene flow (Thawornwattana *et al.*, 2023). The resulting initial model of gene flow is shown in Figure 1a.

We then applied the Bayesian test of gene flow (Ji *et al.*, 2023) to determine the significance of the gene-flow events in the model. While Ji *et al.* (2023) sequentially added introgression events onto the species tree, starting from the most significant introgression events, we fitted the full model with all gene-flow events and used the Bayes factor to remove events that are not strongly supported by the data. The Bayes factor $B_{10}$, in support of the alternative model of gene flow ($H_{1}$) against the null model of no gene flow ($H_{0}$), was calculated via the Savage–Dickey density ratio using an MCMC sample under the $H_{1}$ model (Ji *et al.*, 2023). Gene flow was accommodated using either the MSC-I or MSC-M models. Under MSC-I, the strength of gene flow is measured by the introgression probability, $\varphi_{XY}$, which is the proportion of immigrants in the recipient population Y from X. We defined a "null interval" for the introgression probability, φ<ε, which is a small interval in the parameter space of $H_{1}$ that represents $H_{0}$. Then, $B_{10}$ is approximated by


$$\begin{array}{ccc} B_{10,\varepsilon }=\frac{\mathbb{P}(\varphi <\varepsilon )}{\mathbb{P}(\varphi <\varepsilon |X)}, & & \end{array}$$ (1)

where ℙ(φ<ε) and ℙ(φ<ε|X) are the prior and posterior probabilities for φ<ε, respectively. When ε→0, $B_{10,\varepsilon }\to B_{10}$ (Ji *et al.*, 2023). We used ε=0.01 and confirmed that use of ε=0.001 gave similar results. We used a cut-off of 100. Thus, $B_{10}>100$ means strong support for $H_{1}$ and rejection of $H_{0}$, which is similar to significance at the 1% level in hypothesis testing. $B_{10}<0.01$ means strong support for $H_{0}$ and rejection of $H_{1}$. This does not have an equivalence in hypothesis testing as hypothesis testing can never reject $H_{1}$ with great force. See Ji *et al.* (2023) for detailed discussions.

Under the MSC-M model, the population migration rate, $M_{XY}=m_{XY}N_{Y}$, is defined as the expected number of migrants from the donor species X to the recipient species Y per generation, where $m_{XY}$ is the proportion of migrants in Y from X and $N_{Y}$ is the (effective) population size of species Y. Bayes factor $B_{10}$ in support of $H_{1}:M>0$ against the null $H_{0}:M=0$ was calculated by defining a null interval M<ε, with ε=0.01 or 0.001.

Thus, calculation of $B_{10}$ using eq. 1 requires running the MCMC under the model of gene flow ($H_{1}$). Under the MSC-I, the introgression probability was assigned the prior φ∼ beta(1,1) or U(0,1). We used the option thetamodel = linked-msci in bpp, which assumes the same population-size parameter θ for a branch before and after an introgression event (Ji *et al.*, 2023, Fig. 3b). Under the MSC-M, the migration rate was assigned the gamma prior M∼ G(2,10), with mean 0.2. We used a burn-in of $10^{5}$ iterations, after which we took $5\times 10^{5}$ samples, sampling every 2 iterations. Each analysis was conducted four times to confirm convergence, indicated by the difference in the posterior probability for the *maximum a posteriori* tree between runs being less than 0.3 (Thawornwattana *et al.*, 2022). Runs that did not converge were discarded before the MCMC samples from multiple runs were combined to produce posterior summaries. Each MSC-I run took ∼90hrs using two threads, while each MSC-M run took ∼120 h using four threads.

Gene-flow events that passed the Bayesian test (with $B_{10}>100$) are retained in the final model, which is then used to estimate population parameters, including the rates of gene flow (φ or M), species split times, and population sizes for extant and extinct species on the species tree.

### Assessing the Impact of Taxon Sampling

The evidence for gene flow involving *D. lowei* (see Fig. 1a) appeared to depend on the choice of the outgroup species and on other species included in the dataset. We thus constructed three triplet datasets and three quintet datasets, to assess the impact of taxon sampling. We focussed on gene flow between *D. lowei* and *D. affinis*, for which the evidence is significant in 2 out of 3 triplets in the analysis of Suvorov *et al.* (2022, Table 1).

For the triplet datasets, the species tree was ((*X*, *D. lowei*), *D. affinis*), where *X* was *D. pseudoobscura*, *D. persimilis*, or *D. miranda* (Fig. 1a). The data were also analyzed using summary-based tests (DCT, BLT, and QuIBL), with *D. guanche* used as the outgroup. For the quintet datasets, we included two outgroup species: *D. guanche* and *D. obscura*, so that the species tree was (((*X*, *D. lowei*), *D. affinis*), (*D. obscura*, and *D. guanche*)), where *X* again was one of *D. pseudoobscura*, *D. persimilis*, or *D. miranda* (Fig. 1a). We applied the Bayesian test to assess the evidence for gene flow between *D. lowei* and *D. affinis*.

### Simulating Data to Evaluate Bayesian and Summary Methods for Inferring GeneFlow

As our reanalysis of the *Drosophila* data (for clade 2) produced different results from those of Suvorov *et al.* (2022), we simulated data under the MSC model with gene flow to examine the accuracy of bpp estimation of parameters (Flouri *et al.*, 2020, 2023) and the power of Bayesian test of gene flow (Ji *et al.*, 2023), in comparison with the summary methods used by Suvorov *et al.* (2022).

We conducted two sets of simulations. In the first set, we simulated two datasets using parameter estimates obtained from the *Drosophila* data with the *D. insularis* outgroup under our final MSC-I and MSC-M models with the w→z and x→y gene-flow events, with parameter values given in Table S2 (first half) and Table S3 (first half). Each dataset consisted of 1388 loci, as in the original data halves. The simulate option in bpp (Flouri *et al.*, 2018) was used to generate data under the JC mutation model (Jukes and Cantor, 1969), which were then analyzed using bpp under the same model.

**Fig 2:**
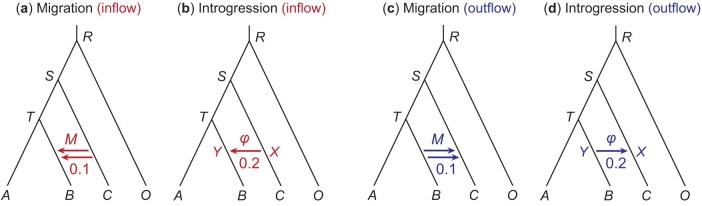
Migration (MSC-M) and introgression (MSC-I) models used to simulate and analyze multilocus sequence data. In the inflow models (**a** and **b**), gene flow is from C→B, whereas in the outflow models (**c** and **d**), it is from B→C. In the MSC-M model (**a** and **c**), migration occurs at the rate of M=0.1 migrants per generation, whereas in the MSC-I model (**b** and **d**), the introgression probability is φ = 0.2. Species divergence times are $\tau_{R}=3\theta$, $\tau_{S}=2\theta$, and $\tau_{T}=\theta$. The introgression time under MSC-I is $\tau_{X}=\tau_{Y}=\theta ∕2$. Two values are used for the population size parameter: θ=0.0025 and 0.01. Each simulated dataset is analyzed using bpp under both the MSC-M and MSC-I models, generating eight simulation-analysis combinations.

In the second set of simulations, we used four artificial MSC-M and MSC-I models for four species (A,B,C, and outgroup O) of Figure 2, with gene flow between nonsister lineages to examine the performance of Bayesian test of gene flow and Bayesian estimation of the rate of gene flow, in comparison with summary methods. The four models of gene flow in Figure 2 were used to simulate gene trees for the loci, which were then used to "evolve" sequences under JC, resulting in a sequence alignment at each locus. The species divergence times were $\tau_{R}=3\theta$, $\tau_{S}=2\theta$, and $\tau_{T}=\theta$, with θ=0.0025 and 0.01. The migration rate was M=0.1 under the MSC-M model (Fig. 2a and c). Under the MSC-I model, the introgression time was $\tau_{X}=\tau_{Y}=0.5\theta$, and the introgression probability was φ=0.2 (Fig. 2b and d).

We examined the effects of the number of loci (L=250,1000,4000), the number of sequences per species per locus (S=2,8), the sequence length (n=250,1000), and mutation rate (θ=0.0025,0.01). As the divergence times (τs) are proportional to θ in our experiment design, the two values of θ mimic genomic regions with different mutation rates (such as coding versus noncoding regions of the genome). We did not run bpp over the large datasets with L=4000 loci and S=8 sequences per species per locus, as those runs were expensive and bpp already achieved 100% power and highly precise parameter estimates in much smaller datasets. One hundred replicates were generated for each parameter setting, with a total of 2000 (=3×2×2×2×100−−400) datasets generated for each of the four models of Figure 2.

Each replicate dataset (simulated under either MSC-I or MSC-M) was analyzed using bpp under both the MSC-M and MSC-I models, resulting in eight simulation-analysis settings. When the data were analyzed, the correct source and donor populations were assumed in the MSC model with gene flow. Gamma priors were assigned to the population size parameters (θ) and the age of the species-tree root ($\tau_{R}$). We used the shape parameter α=2 and adjusted the rate parameter (β) so that the prior means are equal to the true values. For example, for data simulated using θ=0.0025 in the M-M and M-I settings, we used the priors θ∼ G(2,800) and $\tau_{0}∼$ G(2,266), whilst for data simulated using θ=0.01, we used θ∼ G(2,200) and $\tau_{0}∼$ G(2,66). Note that while the same θ was used for all populations when data were simulated, each branch on the species tree had its own θ when the data were analyzed. Under the MSC-I model, we used the thetamodel = linked-msci option so that the same population size parameter is assumed for a branch before and after introgression. Additionally, we used the priors φ∼ beta(1,1) under MSC-I and M∼ G(2,20) under MSC-M. A burn-in of 40,000 iterations was used, after which we took $10^{5}$ samples sampling every 2 iterations.

We evaluated both the power of Bayesian test of gene flow (using the Bayes factor calculated via the Savage-Dickey density ratio; see description above) and Bayesian estimation of parameters, including the rate of gene flow (φ in MSC-I and M in MSC-M). Performance in parameter estimation was measured using the width of the 95% highest probability density (HPD) credible interval (CI).

The simulated quartet data were also analyzed using several summary methods, including those used by Suvorov *et al.* (2022). We assessed both the power to detect introgression and the bias and precision in estimation of the introgression probability. Methods used for testing introgression included HyDe (Blischak *et al.*, 2018), QuIBL (Edelman *et al.*, 2019), DCT (Suvorov *et al.*, 2022), and BLT (Suvorov *et al.*, 2022). Note that those methods are uninformative about the mode of gene flow (whether it occurs in a pulse or over an extended time period), and about the direction of gene flow, whilst bpp assumes a fully specified parametric model. Methods for estimating the introgression probability included HyDe, QuIBL, DCT, and SNaQ (Solis-Lemus and Ane, 2016). Those methods generate only point estimates of φ, while bpp provides in addition a measure of uncertainty in the posterior CIs.

HyDe was implemented using the python script run_hyde.py from Blischak *et al.* (2018) (https://github.com/pblischak/HyDe), which uses a concatenated alignment to count site patterns across all loci. DCT/BLT was implemented using blt_dct_test.r from Suvorov *et al.* (2022) (https://github.com/SchriderLab/Drosophila_phylogeny). QuIBL was run using QuIBL.py from Edelman *et al.* (2019) (https://github.com/miriammiyagi/QuIBL). For SNaQ, we used the PhyloNetworks package (Solis-Lemus and Ane, 2016) to estimate the introgression probability.

QuIBL, DCT, BLT, and SNaQ were applied using gene trees for the individual loci reconstructed by RAxML with default settings (Stamatakis, 2014). Like SNaQ, DCT estimates the introgression probability using inferred gene tree topologies (Suvorov *et al.*, 2022):


$$\hat{\varphi }=\frac{c_{\text{dis2}}–c_{\text{dis1}}}{c_{\text{con}}+c_{\text{dis1}}+c_{\text{dis2}}},$$

where $c_{\text{con}},c_{\text{dis1}},c_{\text{dis2}}$ are the counts of concordant and discordant gene trees, with $\hat{\varphi }=0$ if $c_{\text{dis1}}>c_{\text{dis2}}$.

QuIBL, DCT, and BLT do not allow multiple sequences per species per locus. Thus, input gene trees were constructed using a single sequence chosen at random from the two or eight sequences simulated for each species. Results were similar when different sequences were sampled.

## Results

### Inference of Species Tree and Construction of an Initial Model of Gene Flow forthe *Drosophila* Data

Protein-coding genes from the 11 species in clade 2 of the *Drosophila* phylogeny of Suvorov *et al.* (2022) (Fig. 1a) were separated into two random subsets, with 1389 and 1388 loci, respectively. They were analyzed separately using bpp to estimate the species tree under the MSC model with no gene flow (Yang, 2015; Flouri *et al.*, 2018). Analysis of the two data halves allowed us to assess the robustness of our results to the sampling of loci and also reduced the computational load. All runs across the two halves produced the same species tree topology as inferred by Suvorov *et al.* (2022). We thus concluded that the species phylogeny was well established. The bpp analysis also produced Bayesian estimates of parameters including species divergence times (τ). This information was used, in conjunction with the introgression events inferred by Suvorov *et al.* (2022) in their analyses of triplet and quartet data, to construct an initial model of gene flow for clade 2.

Suvorov *et al.* (2022, Figure 3) inferred three introgression events for clade 2 (Fig. S1). These were, with nodes and branches labeled as in Figure 1a: (i) between x and y, (ii) between branches be and af, and (iii) between lineages bd and *D. lowei*. Event ii had only weak support, with significant evidence for gene flow in only 2 out of 40 feasible triplets (Fig. S1). This was thus discarded in our initial model. Event iii involved branch bd and the *D. lowei* lineage (Fig. 1a), inferred by Suvorov *et al.* (2022) using the f-branch approach (Malinsky *et al.*, 2018). These two lineages did not appear to overlap in time according to bpp estimates of species divergence times. While such a scenario could be interpreted as introgression involving an extinct or unsampled “ghost” lineage (e.g., Yang and Flouri, 2022, Fig. 9a–c), we note that the introgression event was not well supported by the DCT/BLT triplet tests of Suvorov *et al.* (2022, Fig. 3). Those tests supported introgression between *D. lowei* and *D. affinis* and between *D. lowei* and *D. azteca*, but not between *D. lowei* and *D. athabasca* (Table S1). Thus, we replaced event iii by two events involving the daughter branches, between *D. lowei* and *D. affinis* (or p↔q) and between *D. lowei* and *D. azteca* (or u↔v) (Fig. 1a).

Our initial model of gene flow for clade 2 thus involved three gene-flow events: one ancestral and two involving extant taxa (Fig. 1a). As the triplet methods used by Suvorov *et al.* (2022) are agnostic about the direction of gene flow, we treated each event as a bidirectional gene-flow event. This way of determining the direction of gene flow involves a computational cost but was found to work well in simulations (Thawornwattana *et al.*, 2023). We fitted both the MSC-I and MSC-M models of gene flow. Two variants of the MSC-I model were considered, which differed in the time order of the two introgression events involving *D. lowei* (u↔v and p↔q). The results are summarized in Table 1 for MSC-I and Table 2 for MSC-M.

**Table 1:** Posterior means and 95% HPD CIs (in parentheses) of introgression probabilities (φ), introgression times (τ), and Bayes factors in support of gene flow ($B_{10}$) in the bpp analysis of the *Drosophila* data under the MSC-I models of Figure 1

	First half (1389 loci)			Second half (1388 loci)		
Introgression	$\hat{\varphi }$	$\hat{\tau }$	$B_{10}$	$\hat{\varphi }$	$\hat{\tau }$	$B_{10}$
Model 1a: *D. lowei* ↔*D. affinis* introgression first (Fig. 1a)						
rb→ac (or y→x)	0.0014 (0.0000, 0.0041)	0.0261 (0.0257, 0.0265)	0.01	0.0011 (0.0000, 0.0032)	0.0260 (0.0256, 0.0265)	0.01
ac→rb (or x→y)	0.0980 (0.0787, 0.1173)		∞	0.0917 (0.0756, 0.1081)		∞
*D. lowei* →*D. azteca*	0.0027 (0.0003, 0.0058)	0.0032 (0.0001, 0.0057)	0.01	0.0009 (0.0000, 0.0026)	0.0025 (0.0000, 0.0053)	0.01
*D. azteca* →*D. lowei*	0.0008 (0.0000, 0.0024)		0.01	0.0010 (0.0000, 0.0030)		0.01
*D. lowei* →*D. affinis*	0.0026 (0.0000, 0.0062)	0.0050 (0.0025, 0.0066)	0.01	0.0011 (0.0000, 0.0035)	0.0047 (0.0020, 0.0064)	0.01
*D. affinis* →*D. lowei*	0.0008 (0.0000, 0.0024)		0.01	0.0016 (0.0000, 0.0040)		0.01
Model 1b: *D. lowei* ↔*D. azteca* introgression first (Fig. 1a)						
rb→ac (or y→x)	0.0014 (0.0000, 0.0041)	0.0261 (0.0257, 0.0265)	0.01	0.0011 (0.0000, 0.0032)	0.0260 (0.0255, 0.0264)	0.01
ac→rb (or x→y)	0.0980 (0.0788, 0.1173)		∞	0.0920 (0.0758, 0.1085)		∞
*D. lowei* →*D. azteca*	0.0026 (0.0001, 0.0058)	0.0063 (0.0032, 0.0102)	0.01	0.0010 (0.0000, 0.0029)	0.0075 (0.0034, 0.0103)	0.01
*D. azteca* →*D. lowei*	0.0008 (0.0000, 0.0025)		0.01	0.0014 (0.0000, 0.0039)		0.01
*D. lowei* →*D. affinis*	0.0020 (0.0000, 0.0051)	0.0033 (0.0001, 0.0061)	0.01	0.0011 (0.0000, 0.0033)	0.0034 (0.0028, 0.0062)	0.01
*D. affinis* →*D. lowei*	0.0008 (0.0000, 0.0024)		0.01	0.0013 (0.0000, 0.0036)		0.01
Model 2: final model with unidirectional introgression from w→z and x→y (Fig. 1b)						
ra→rb (or w→z)	0.7275 (0.6893, 0.7690)	0.0381 (0.0375, 0.0387)	∞	0.7124 (0.6806, 0.7432)	0.0388 (0.0382, 0.0394)	∞
ac→rb (or x→y)	0.0688 (0.0546, 0.0832)	0.0257 (0.0253, 0.0261)	∞	0.0690 (0.0561, 0.0822)	0.0257 (0.0253, 0.0261)	∞

Note: Initial models 1a & 1b differ in the time order of two bidirectional introgression events: *D. lowei*  ↔*D. azteca* versus *D. lowei*  ↔*D. affinis* (Fig. 1a). As the time of the ac→rb introgression ($\tau_{x\to y}$) was very close to the species divergence time $\tau_{a}$ (Fig. 1a), the introgression event was moved to the parental branch in (w→z, Fig. 1b), but there was support in the data for the x→y introgression, so that both events were included in the final model 2. Bayes factor for testing introgression ($B_{10}$) was calculated using the Savage–Dickey density ratio with ε=0.01 (Ji *et al.*, 2023). $B_{10}=\infty$ occurs when there are no MCMC samples with φ<ε=0.01, whereas $B_{10}=0.01$ occurs when all MCMC samples have φ<ε.

**Table 2:** Posterior means and 95% HPD CIs (in parentheses) of migration rates (M) and Bayes factors ($B_{10}$) in the bpp analysis of the *Drosophila* data under the MSC-M model of Figure 1

	First half (1389 loci)		Second half (1388 loci)	
Migration	$\hat{M}$	$B_{10}$	$\hat{M}$	$B_{10}$
Model 1 (Fig. 1a)				
rb→ac (or y→x)	0.0220 (0.0028, 0.0449)	0.01	0.0075 (0.0002, 0.0181)	0.01
ac→rb (or x→y)	0.3065 (0.2255, 0.3940)	∞	0.3375 (0.2588, 0.4212)	∞
*D. lowei* →*D. azteca*	0.0108 (0.0014, 0.0238)	0.00	0.0084 (0.0004, 0.0215)	0.01
*D. azteca* →*D. lowei*	0.0142 (0.0018, 0.0293)	0.01	0.0143 (0.0015, 0.0333)	0.02
*D. lowei* →*D. affinis*	0.0134 (0.0011, 0.0306)	0.00	0.0171 (0.0020, 0.0384)	0.00
*D. affinis* →*D. lowei*	0.0148 (0.0012, 0.0316)	0.02	0.0181 (0.0032, 0.0378)	0.01
Model 2: final model with unidirectional migration from w→z and x→y (Fig. 1b)				
ra→rb (or w→z)	0.5677 (0.5183, 0.6151)	∞	0.6031 (0.5546, 0.6523)	∞
ac→rb (or x→y)	0.0111 (0.0003, 0.0253)	0.01	0.0090 (0.0002, 0.0204)	0.01

Note: Model 1 assumes three bidirectional migration events or three pairs of migration rates (Fig. 1a). All of them were rejected except $M_{x\to y}$ based on the Bayes factor ($B_{10}$). In the final model 2, we added the w→z migration, similarly to analysis under the MSC-I model (Table 1).

Under the MSC-I model, introgression from branches ac to rb (or x→y, Fig. 1a) had the strongest signal. The estimated introgression probability was ${\hat{\varphi }}_{x\to y}=0.098$ and 0.092 for the two data halves, while the Bayes factor $B_{10}=\infty$ for the Bayesian test (Table 1, models 1a and 1b). Introgression in the opposite direction (y→x) was found to be absent, with the model of introgression rejected strongly ($B_{10}\leq 0.01$). Apart from the x→y introgression, all other introgression events were rejected at the $B_{10}\leq 0.01$ cut-off (Table 1). Note that the Bayesian test may strongly favor the null model and reject the more general model of gene flow, unlike hypothesis testing, which may fail to reject the null hypothesis but may never support it strongly.

The MSC-M model produced results consistent with the MSC-I model (Table 2, model 1). Similarly the only gene-flow event supported was from x→y, with the estimated rate to be $M_{x\to y}=0.31$ and 0.34 migrants per generation for the two halves, respectively, while gene flow in the opposite direction was found to be absent. Also the x→y migration was the only one that was significant ($B_{10}>100$), while all other gene-flow events were rejected by the Bayesian test at the $B_{10}\leq 0.01$ cut-off.

Interestingly, the time of the x→y introgression under the MSC-I model was nearly identical to the divergence time at the mother node a (Fig. 1a): ${\hat{\tau }}_{x}={\hat{\tau }}_{y}=0.0261$ (with the 95% HPD CI 0.0257–0.0265) and 0.260 (0.0256–0.0265) for the two halves, respectively, compared with ${\hat{\tau }}_{a}=0.0261$ (0.0257–0.0265) and 0.0261 (0.0256–0.0265) under models 1a and 1b of Table 1. This may suggest that the introgression event was assigned to the wrong branch in the initial model; Huang *et al.* (2022a) found that when introgression is incorrectly assigned onto a daughter or mother branch of the lineage genuinely involved in gene flow, the introgression time tends to get stuck on the species divergence time. Thus, we considered a model in which the x→y introgression was replaced by introgression involving the parental branch (w→z). This model produced greater estimates of the introgression probability, $\varphi_{w\to z}=0.248$ (CI 0.207–0.291) for the first half and 0.393 (0.349–0.440) for the second half, and with the introgression time away from the species divergence time.

We also fitted an MSC-I model with both w→z and x→z introgressions (Fig. 1b), with the expectation that introgression event that did not occur should have low estimated rates, rejected by the test (Huang *et al.*, 2022a; Thawornwattana *et al.*, 2022). The analysis detected very strong evidence for gene flow between the sister lineages, with ${\hat{\varphi }}_{w\to z}=0.728$ (0.689–0.769) and 0.712 (0.681–0.743) for the two data halves (Table 1, model 2). The evidence for the x→y introgression was also significant although the rate was much lower, at ${\hat{\varphi }}_{x\to y}=0.069$ (0.055–0.083) and 0.069 (0.056–0.082) (Table 1, model 2).

We further assessed possible impacts of including an outgroup species, using either *D. melanogaster* or *D. insularis* as the outgroup, besides the 11 ingroup species in clade 2 (Tables S2 and S3). Some parameters such as the population size for the root of the species tree are known to be sensitive to the inclusion of outgroup species (Burgess and Yang, 2008). The introgression probabilities ($\varphi_{w\to z},\varphi_{x\to y}$) and introgression times ($\tau_{w}=\tau_{z},\tau_{x}=\tau_{y}$) are very similar among the datasets (for two halves and two outgroups) (Tables S2 and S3), and also similar to the estimates without the outgroup (Table S4). Estimates of $\theta_{r}$ varied depending on the outgroup used (Tables S2 and S3), possibly because branch r ancestral to clade 2 represents different populations depending on the outgroup.

Given the introgression events between extant species inferred using triplet summary methods (Suvorov *et al.*, 2022), we fitted MSC-I models incorporating various introgression events between extant species, when the w→z and x→y introgression events are already accommodated in the model (Table S5). In particular, we tested bidirectional introgression events involving *D. lowei* (Fig. 1a). All gene-flow events involving extant species, including bidirectional introgression events involving *D. lowei*, were rejected, with $B_{10}\leq 0.01$ (Table S5). The w→z and x→y introgressions remained the only significant events, and parameter estimates were virtually identical to those under model 2 with the w→z and x→y introgressions only (Table S4). We examine the impact of taxon sampling on inference of gene flow below.

In the MSC-M model, we also included the w→z migration in addition to the x→y migration (Table 2, model 2). Similarly we obtained high estimates of migration rate between the sister lineages, $M_{w\to z}=0.568$ (CI 0.518–0.615) and 0.603 (0.555–0.652) immigrants per generation, and the Bayesian test was highly significant. The migration rate for the nonsister lineages was much lower, estimated to be ${\hat{M}}_{x\to y}=0.011$ and 0.009 for the two halves, and was not significant according to the test. Thus, the evidence for the x→y gene flow was inconsistent between the MSC-I and MSC-M models. This could be due to weak signal or low information content in the data, or lower power of the MSC-M model than the MSC-I model (Thawornwattana *et al.*, 2024).

By integrating all analyses above, we suggest model 2 of Figure 1b as our final inferred model for clade 2 on the *Drosophila* phylogeny (Suvorov *et al.*, 2022), which includes both the x→y and w→z introgression events.

### Estimation of Model Parameters on the *Drosophila* Species Tree

We fitted the final model of Figure 1b to estimate model parameters, with gene flow accommodated using either the MSC-I or the MSC-M models. Estimates of the rate of gene flow (φ in MSC-I and M in MSC-M) are given in Tables 1 and 2 (model 2), while those for all parameters are in Table S4.

As discussed in the section above, the estimated rate of gene flow between the sister lineages (w→z) was very high under both the MSC-I and MSC-M models (Tables 1, model 2 and Table 2, model 2). In comparison, the estimated rate of x→y gene flow was much lower and was indeed not significant under the MSC-M model. Here, we ask whether the two models recover similar amounts of gene flow between the sister lineages. If the MSC-M model is the true model with the w→z migration occurring over a time period Δτ, the expected cumulative proportion of migrants in the recipient population z will be


$$\begin{array}{ccc} \varphi_{0}=1-e^{-4M_{wz}\Delta \tau ∕\theta_{z}} & & \end{array}$$ (2)

(Huang *et al.*, 2022a). Using the estimates under MSC-M (Table S4), we calculated the expected introgression probability for the MSC-I model to be $\varphi_{0}=1-e^{-4\times 0.568\times (0.0748-0.0260)∕0.0692}=0.798$ for the first half, and 0.789 for the second half, compared with the estimates under the MSC-I: 0.728 and 0.712. The estimates are similar, with slightly more gene flow inferred under MSC-M than under MSC-I.

**Fig 3:**
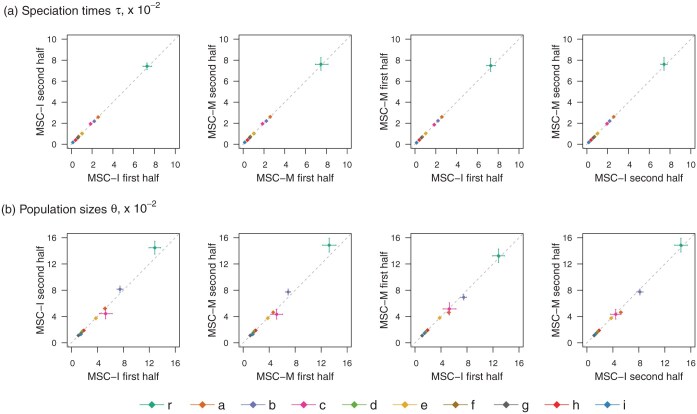
Posterior means and 95% HPD CIs for a) species divergence times (τ, mutations per site) and b) population sizes (θ) in the final model of Figure 1b obtained from bpp analyses of the *Drosophila* data under the MSC-I and MSC-M models.

Estimates of species divergence times (τ) and population sizes (θ) for the two data halves under the MSC-I and MSC-M models are shown in Figure 3. The four data-model combinations produced nearly identical estimates. Estimates of the age of the root for the clade ($\tau_{r}$) differ considerably depending on whether gene flow is accommodated in the model (cf. Figs 1a and 1b). This is consistent with previous studies which have shown that ignoring gene flow between species leads to serious underestimation of species split times (Leaché *et al.*, 2014; Tiley *et al.*, 2023; Thawornwattana *et al.*, 2023).

For both data halves, $\hat{\varphi }>\frac{1}{2}$ under MSC-I, so that the majority of the lineages from descendent species of node a (i.e., *D. subobscura, D. guanche, D. obscura, D. bifasciata*) are traced back to the introgression branch rz rather than the speciation branch rw (Fig. 1b). This is also the prediction of the MSC-M model since the estimates suggest $\varphi_{0}>\frac{1}{2}$ by eq. 2. We also note that $\tau_{r}$ in model 1 (Fig. 1a) was similar to $\tau_{w}=\tau_{z}$ in model 2 (Fig. 1b). Thus, the histories of sequence divergences reflected in the gene trees predicted by the two models (one with the x→y gene flow only and the other with both x→y and w→z gene flow) are somewhat similar.

Our analysis using bpp assumed the JC model (Jukes and Cantor, 1969). To see whether the mutation/substitution model affects the results, we analyzed the data under the final MSC-I and MSC-M models of Figure 1**b** assuming the GTR mutation model (Tavaré, 1986; Yang, 1994) instead of JC (Fig. S2, Table S6). The estimates under the JC and GTR models were very similar, and the mutation model had little effects. Estimates of introgression probabilities and migration rates were also very similar between the two models (Table S6). This robustness to the mutation model is expected because the main role of the mutation model in bpp analyses is to correct for multiple hits at the same site. As the sequence data from closely related species are extremely similar, any mutation model including the infinite-sites model (Takahata *et al.*, 1995) should work well. Similar observations were made by Shi and Yang (2018) and Flouri *et al.* (2022).

### The Impact of Taxon Sampling on Inference of Gene Flow Involving *D.lowei*

While there was significant evidence for gene flow between *D. lowei* and either *D. affinis* or *D. azteca* in the DCT/BLT tests of Suvorov *et al.* (2022, data S2), those gene-flow events were rejected in our analyses of data including all species in the group (Table 1). We thus examined the impact of taxon sampling, by constructing three triplet datasets and three quintet datasets and analyzing them using bpp. We focus on gene flow between *D. lowei* and *D. affinis*, for which the evidence was significant in two out of three triplets in the analysis of Suvorov *et al.* (2022, data S2).

First, we analyzed the triplet datasets using QuIBL and DCT/BLT to examine the impact of the outgroup species. The assumed ingroup tree was ((*X*, *D. lowei*), *D. affinis*), where *X* was *D. pseudoobscura*, *D. persimilis*, or *D. miranda*, while *D. guanche* was used as the outgroup (Fig. 1a). Unrooted quartet trees were generated using RAxML under the JC model, rooted with the outgroup, and then used as input for DCT/BLT and QuIBL. All summary-based tests were significant for all triplets. Suvorov *et al.* (2022) used *Anopheles gambiae* as the outgroup, and inferred quartet gene trees under the GTR+I+G model, finding that BLT and QuIBL were significant for all three triplets, while DCT was significant in two out of three triplets. The *Anopheles* outgroup is very distantly related to the ingroup species, and a closely related outgroup may be preferable as long as it is not involved in hybridization with the ingroup species. Nevertheless, the results from the summary methods are consistent between the two studies.

Next, we analyzed the triplet datasets using bpp (Table S7). Bidirectional introgression between *D. lowei* and *D. affinis* was specified in the MSC-I model. In all three datasets, there was strong evidence for introgression from *D. lowei*  →  *D. affinis*, with $B_{10}>100$ and the estimated introgression probability ${\hat{\varphi }}_{p\to q}=$ 4.2–4.8%. There was also strong evidence rejecting introgression in the opposite direction (with $B_{10}\leq 0.01$ and ${\hat{\varphi }}_{q\to p}\approx 0.00$). Thus, the bpp analysis of triplet datasets is consistent with the summary methods (DCT/BLT), although bpp was able to infer the direction and strength of gene flow, rejecting the q→p introgression.

Finally, the quintet datasets which include two outgroup species, *D. guanche* and *D. obscura*, were analyzed using bpp under MSC-I assuming bidirectional introgression between *D. lowei* and *D. affinis*, either with or without accommodating the w→z and x→y introgressions (Fig. 1b, Table S7). In all cases, the q→p introgression was rejected, as in the analysis of the triplet data. Without the w→z and x→y introgressions in the model, the p→q introgression rate was low (1–2%) and was not significant (with $B_{10}<100$ in all three datasets). When the w→z and x→y introgressions were assumed in the model, the p→q introgression became significant in all three quintet datasets (with $B_{10}>100$), with ${\hat{\varphi }}_{p\to q}\approx$ 4.1–5.7% (Table S7). We also note that in the analysis of data from all 11 species in clade 2, under the model which incorporates the w→z and x→y introgressions, the estimated introgression probability $\varphi_{p\to q}$ was very low (0.1–0.2%) and was rejected with $B_{10}\leq 0.01$ (Table S5, last section).

In summary, while the q→p introgression was rejected in all analyses, the Bayesian test of the p→q introgression was sensitive to the species included in the data and to whether other major introgression events (w→z, x→y) were already accounted for in the model. The reasons for this sensitivity are not well-understood. We suspect that part of the difficulty may be due to problems of sampling, as the data consist of only one sequence per species per locus. The introgression probability is defined as a proportion of migrants in the recipient species. Knowledge of the population size or genetic diversity of the recipient species should help our inference of the contribution to that diversity from introgression. We note that the population size parameters $\theta_{\mathrm{D. lower}}=\theta_{p}$ and $\theta_{\mathrm{D. affinis}}=\theta_{q}$ are very poorly estimated with wide CIs, and the introgression probability $\varphi_{p\to q}$, if nonzero, was relatively low (<6%) (Table S7), so that inference may be easily affected by factors other than gene flow. Including multiple samples per species may be expected to increase the information in the data about the p→q introgression (see Discussion).

### Analyses of Simulated Data by Bayesian and Summary Methods: *Drosophila*-Based Simulation

Our Bayesian analysis of the *Drosophila* clade-2 data of Suvorov *et al.* (2022) produced different results from those obtained by Suvorov *et al.* (2022) using triplet methods. To understand possible reasons for the differences, we conducted two sets of simulations to study the statistical behaviors of the methods.

In the *Drosophila*-based simulation, we used parameter estimates of Tables S2 (first half) and S3 (first half) obtained from the bpp analysis of the clade-2 data including the *D. insularis* outgroup under the final MSC-I and MSC-M models with the w→z and x→y gene-flow events. Two data halves, each of 1388 loci, were simulated. Bayesian estimates of parameters (Table S8) were very close to the true parameter values, and the 95% HPD CIs were similar to those in the analysis of the real data (cf: Table S4).

In the bpp analyses, we used diffuse gamma priors on parameters τ and θ with the prior means matching the true values (the 1x priors): $\tau_{r}∼$ G(2,50) and θ∼ G(2,200). To assess the impact of the priors, we varied the prior means to be either 10 times larger (the 10x priors): $\tau_{r}∼$ G(2,5) and θ∼ G(2,20), or 10 times smaller (the 0.1x priors): $\tau_{r}∼$ G(2,500) and θ∼ G(2,2000). The priors had little impact on estimation of the species split times, but some population size parameters were somewhat affected, with the use of the 0.1x priors causing underestimation of $\theta_{r}$ and $\theta_{c}$ (Fig. S3). Estimates of introgression probabilities (φ) and migration rates (M) were very close to the true values (Table S9). Overall, the posterior was robust to such orders-of-magnitude changes to the prior mean, apparently because the datasets analyzed in this study were large.

Note that the major introgression event in the true model, from w→z, is between sister lineages and is thus unidentifiable by triplet methods used by Suvorov *et al.* (2022). Instead, we applied DCT (which is based on gene-tree counts) and BLT (which is based on branch lengths) to detect the x→y introgression by constructing triplets. In 8/28 triplets, significant evidence was detected by DCT. No signal was detected by BLT.

### Analyses of Simulated Data by Bayesian and Summary Methods: QuartetData

In the second set of simulations, we used the MSC-M and MSC-I models for four species (A,B,C, and outgroup O) of Figure 2, with gene flow between nonsister lineages (B,C). Divergence times (τ) and population sizes (θ) resemble estimates from the *Drosophila* data, but we used a range of parameter values. Each dataset was analyzed using bpp under both the MSC-M and MSC-I models, resulting in eight simulation-analysis settings. We examine both estimation of model parameters (in particular the rate of gene flow) and Bayesian test for the presence of gene flow. This set of simulation is similar to previous studies that examined the properties of the Bayesian method (Huang *et al.*, 2020, 2022a; Thawornwattana *et al.*, 2023, 2024), but here we included a number of summary methods.

#### Bayesian estimation in quartet data

**Fig 4:**
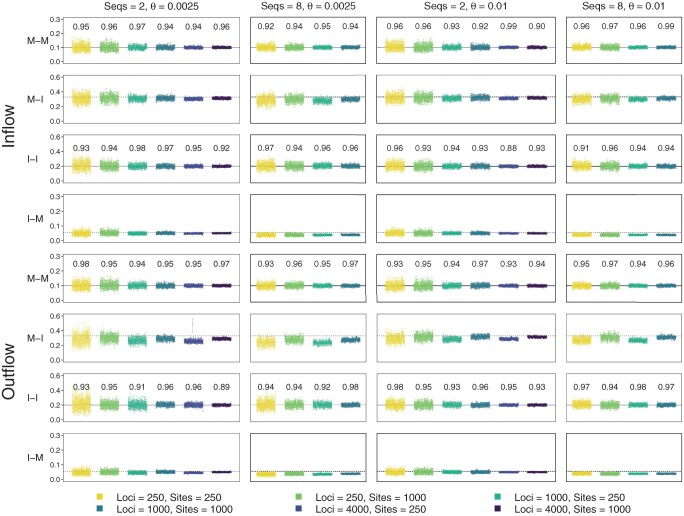
Posterior means and 95% CIs for introgression probabilities (φ) in the MSC-I model and migration rates (M) in the MSC-M model obtained from the bpp analysis of 100 simulated data replicates. Datasets were simulated under the four models of Figure 2 and analyzed under both the MSC-M and MSC-I models, with eight settings in total. For example, in the inflow-M-I setting, replicate datasets were simulated under the inflow-migration (MSC-M) model (Fig. 2a) and analyzed under the introgression (MSC-I) model (Fig. 2b). Results for other parameters in the eight simulation settings are in Figures S4–S11. Numbers above the CI bars represent the CI coverage probability. Solid black lines represent true parameter values. Dashed black lines represent the theoretical expectations when the mode of gene flow is misspecified (eq. 2). Large datasets under settings with L=4000 loci and S=8 sequences per species per locus (with either 250 or 1000 sites) were not analyzed.

Here, we discuss the estimation of the rate of gene flow (φ in MSC-I and M in MSC-M) (Fig. 4), with results for all parameters summarized in Figures S4–S11 and discussed in the SI text. In the M-M and I-I settings (Figs 4), data were simulated and analyzed under the same model. The rate of gene flow was well estimated, with the posterior means around the true values, while the 95% HPD CIs become narrower when the data size increases. In informative datasets, the coverage of the 95% CI was in general >95%. Introgression probability was more precisely estimated in the inflow model (with gene flow from C→B, Fig. 2a and b) than in the outflow model (B→C, Fig. 2c and d) (Figs S6 inflow I-I vs. S10 outflow I-I). For example, the CI width in the least informative data set (L=250,S=2,n=250, θ=0.0025) was ∼43% narrower under the inflow than outflow models. These results are consistent with the observation of Thawornwattana *et al.* (2023).

In the M-I and I-M settings (Fig. 4), the mode of gene flow was misspecified. The analysis of Huang *et al.* (2022a) suggests that when data are generated under MSC-M but analyzed under MSC-I, not all gene flow that has occurred is recoverable, with $\hat{\varphi }<\varphi_{0}$ (eq. 2). This was the case in the simulation here (Figs 4, inflow M-I and outflow M-I). The underestimation was more serious (with larger difference between $\hat{\varphi }$ and $\varphi_{0}$) in the outflow case than in the inflow case.

#### Bayesian test in quartet data

**Fig 5:**
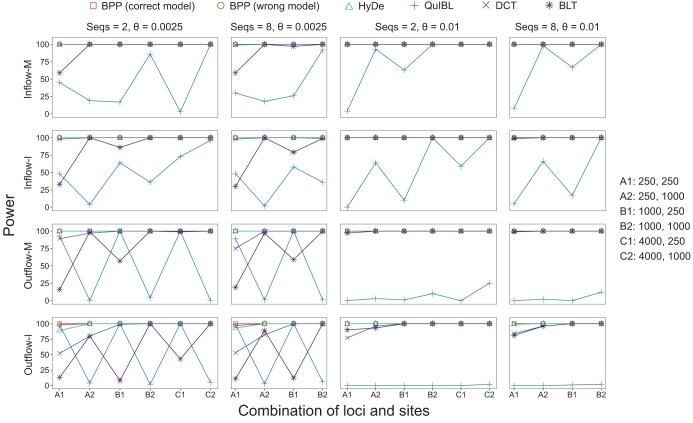
Power (percentage of replicates in which the null model of no gene flow is rejected at the 1% level) of bpp (MSC-I and MSC-M), HyDe, QuIBL, DCT, and BLT to detect gene flow in data simulated under the four models of gene flow in Figure 2. Bayesian test of gene flow using bpp is conducted assuming either the correct model (e.g., Inflow-M-M) or incorrect model (e.g., Inflow-M-I), with gene flow detected if the Bayes factor $B_{10}>100$. Data configurations are specified in the number of loci (L) and the number of sites (n): for example, in configuration “A2: 250, 1000”, each dataset consists of L=250 loci, each of n=1000 sites. Bayesian estimates of parameters from the same data are shown in Figures 4 and S4–S11.

Bayesian test of gene flow overall showed very high power in simulated quartet data (Fig. 5). At the 1% cut-off (i.e., with $B_{10}>100$), the test achieved ∼100% power in all simulation settings. This was the case even in the least informative datasets (with L=250 loci, n=250 sites, and at the low mutation rate with θ=0.0025). In particular, power was ∼100% in the M-I and I-M settings as well, when the mode of gene flow was misspecified. For instance, if gene flow occurred continuously over an extended time period according to the MSC-M model but was assumed to occur in a pulse in the MSC-I model, the test still detected gene flow with nearly full power (Fig. 5, inflow-M and outflow-M, bpp-wrong model).

#### Estimation by summary methods in quartet data

**Fig 6:**
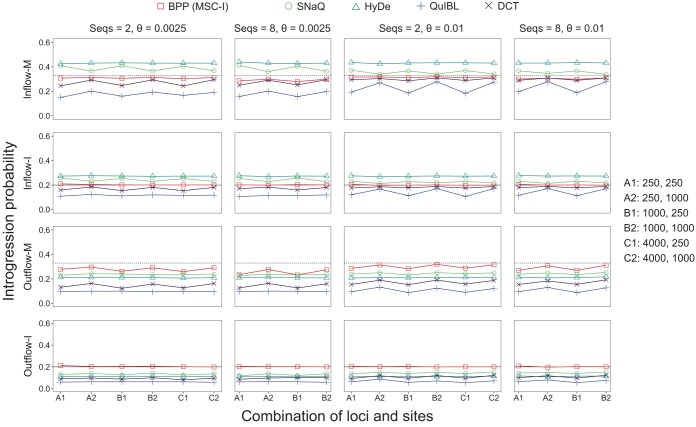
Average estimates of introgression probability (φ) produced by bpp (MSC-I), SNaQ, HyDe, QuIBL, and DCT for each of the four gene-flow models of Figure 2. Black solid lines represent the true value of φ in the MSC-I model, whereas dashed lines represent the expected value $\varphi_{0}$ (eq. 2) when the data are generated under the MSC-M model. See legend to Figure 5.

We applied several summary methods to estimate the introgression probability (Fig. 6) and to test for gene flow (Fig. 5). For data simulated under MSC-I (Fig. 6, inflow-I and outflow-I), all summary methods for estimating φ appeared to be biased. In the inflow scenario, SNaQ and HyDe overestimated the introgression probability, while DCT and QuIBL produced underestimates (Fig. 6, inflow-I). In the outflow scenario, all summary methods produced underestimates (Fig. 6, outflow-I). QuIBL, in particular, produced gross underestimates. This bias of the QuIBL method was noted previously by Edelman *et al.* (2019).

#### Test of gene flow by summary methods in quartet data

Next, we examined the power of summary methods for testing for gene flow, in comparison with bpp (Fig. 5). While bpp achieved ∼100% power in all datasets, even when the mode of gene flow was misspecified, the performance of the summary methods varied. The two methods based on gene-tree branch lengths, QuIBL and BLT, had particularly low power for short sequences (250 sites instead of 1000) and at the low mutation rate (with θ=0.0025 instead of 0.01). This may be expected since short and highly similar sequences contain little phylogenetic information, leading to large sampling errors in the estimated branch lengths, while those errors are ignored by both methods. QuIBL had ∼0% power in data generated under the outflow model. This appeared to be due to the fact that QuIBL assumes a triplet species tree with an inflow model of introgression rather than outflow (Edelman *et al.*, 2019, Figs S61 and S62), so that for those data, the assumed direction of gene flow was incorrect.

HyDe showed good power. As it uses site-pattern counts pooled over loci, it is not sensitive to sampling errors in the estimated gene-tree topology and branch lengths at each locus. We note that HyDe is based on a hybrid-speciation model, which is a special case of the inflow model with symmetry in the population size (Ji *et al.*, 2023). Previously HyDe was found to perform poorly when those assumptions were not met; in particular, HyDe was found to lack power when gene flow occurred in the opposite (outflow) direction (Ji *et al.*, 2023, Figure 9; Pang and Zhang, 2024). In the simulation here, the method performed relatively well (Fig. 5), apparently because the same population size was used for all species in the simulation model, so that the assumptions of HyDe were largely met.

Finally DCT showed low power in the least informative datasets but was not so sensitive to short sequences as were QuIBL and BLT (Fig. 5). This may be because DCT uses gene-tree topologies but not branch lengths.

## Discussion

### Likelihood and Summary Methods for Inferring Gene Flow

Our simulations highlight the desirable statistical properties of the Bayesian method implemented in bpp. The power to detect gene flow via the Bayesian test (Ji *et al.*, 2023) was high, even when the information content of the dataset was low and even if the mode of gene flow was misspecified. Bayesian estimation of parameters including introgression probabilities and migration rates was highly accurate. We found that if the mode of gene flow was misspecified (when the true model was MSC-I and the analysis model was MSC-M, or vice versa), the Bayesian method may underestimate the amount of gene flow. However, the shared parameters between the two models were reliably estimated. The simulation results here are consistent with and extend previous simulations which examined the Frequentist properties of Bayesian test and Bayesian estimation under the MSC model with gene flow (Huang *et al.*, 2020, 2022a; Ji *et al.*, 2023; Thawornwattana *et al.*, 2023; Pang and Zhang, 2024).

The performance of summary methods in the simulation varied considerably (Figs 5 and 6). All summary methods for estimating the introgression probability were found to be biased (Fig. 6). In particular, branch length-based methods such as QuIBL performed poorly and had low power to detect gene flow, except in the most informative inflow datasets. When the species are closely related and the sequences are highly similar, estimated branch lengths in reconstructed gene trees are expected to have considerable errors and uncertainties, which may affect the performance of those methods.

Suvorov *et al.* (2022) has relied on the f-branch approach to integrate results of many triplet analyses. This was designed to move introgression events to ancestral branches on the species tree, as gene flow involving ancestral lineages may show up as significant introgression events in many species triplets, which may be hard to interpret (Malinsky *et al.*, 2018). Disturbingly, a recent study demonstrated that the commonly used triplet methods, such as the D-statistic, HyDe, and SNaQ, do not have the ability to identify different introgression models, including ancestral introgression from an outgroup, and inflow and outflow between nonsister lineages (Pang and Zhang, 2024). It is unclear how the performance of $f_{\text{branch}}$ is affected by such unidentifiability. In general, research is needed to understand the behavior of the approach in realistic scenarios involving multiple introgression events on a species tree of more than three species when test of gene flow is always conducted using species triplets.

Overall analyses of real and simulated data in this study as well as in previous studies (Huang *et al.*, 2020, 2022a; Ji *et al.*, 2023; Thawornwattana *et al.*, 2023; Pang and Zhang, 2024) have highlighted large gaps between full likelihood methods (such as bpp) and summary methods. Summary methods are orders-of-magnitude faster computationally and can easily accommodate genome-scale datasets, while likelihood methods have much better statistical performance (with higher power in inferring gene flow and less bias in estimating its rate). There is an urgent need for improving the statistical properties of summary methods and the computational efficiency of likelihood methods for inferring gene flow using genomic sequence data.

### Gene Flow in *Drosophila*

There has been long-standing interest in gene flow between species on the *Drosophila* phylogeny. Noor *et al.* (2000) analyzed within-species polymorphism and between-species divergence along the genome to infer gene flow between *D. pseudoobscura* and *D. persimilis*. The population genetic analysis did not identify the direction of gene flow. Wang and Hey (2010, see also Dalquen *et al.*, 2017) explicitly modeled the coalescent-with-migration process in the so-called isolation-with-migration (IM) model and used multilocus sequence data to infer low but significant gene flow from *D. simulans* to *D. melanogaster*, with no gene flow in the opposite direction. The study of Suvorov *et al.* (2022) is noteworthy for its use of 155 *Drosophila* genome assemblies, covering the whole *Drosophila* genus and suggesting multiple instances of between-species gene flow.

Our reanalysis of data for clade 2 in the *Drosophila* genus of Suvorov *et al.* (2022) has confirmed the authors’ overall conclusion that gene flow is prevalent on the species phylogeny and extended that work by characterizing the lineages involved in gene flow and its direction and by estimating the timing and rates of gene flow. We inferred a gene-flow event involving sister lineages which is unidentifiable by the triplet summary methods used by Suvorov *et al.* (2022), while some introgression events inferred by Suvorov *et al.* (2022) were rejected in our Bayesian test. Our simulation in general demonstrates the accuracy and robustness of bpp and raised concerns about the reliability of the summary methods used by Suvorov *et al.* (2022). Our analyses suggest a need for a reanalysis of gene flow for the other clades on the *Drosophila* phylogeny.

Here, we note a few limitations with both our Bayesian analysis and the sequence data, which may affect our inference. First, our search in the space of models was not exhaustive. We used the Bayesian test to confirm or remove gene-flow events proposed in the triplet analyses of Suvorov *et al.* (2022), and in some cases repositioned events to ancestral branches when our analysis suggested incorrect placement (Table 1). We also assessed various scenarios of gene flow involving extant species (Table S5). This constitutes a limited search in the space of introgression models. The use of a stringent cut-off for $B_{10}$ in the test may lead to false negatives (i.e., failure to detect gene flow when it exists), but the test appeared to be very powerful in simulations (this study and Ji *et al.*, 2023).

Second, some concerns may be raised about the suitability of the sequence data of Suvorov *et al.* (2022). The data consist of single-copy protein-coding genes compiled to infer the phylogeny and to estimate divergence times for the whole *Drosophila* genus, with divergence times >50MY (or >100MY from the *A. gambiae* outgroup). While single-copy orthologous genes are ideal for phylogenetic reconstruction and divergence time estimation among distantly related species, which are major objectives of the study of Suvorov *et al.* (2022), they may not be optimal for inferring gene flow between closely related species. The data for clade 2 involve a high degree of incompleteness, with missing species at ∼50% of the gene loci. Noncoding parts of the genome tend to have higher mutation rates and may be more informative than conserved exons, even though they may pose challenges to genome assembly. Also the data appear to be “haploid consensus sequences,” with genotypic phase at heterozygous sites in the diploid sequence resolved effectively at random, creating chimeric sequences that may not exist in nature and may impact on genealogy-based analyses under the MSC (Andermann *et al.*, 2019; Huang *et al.*, 2022b). Furthermore, the data consist of only one sample per species per locus. Summary methods considered here do not use information in multiple samples per species, and indeed some authors suggest that “adding more samples provides little new information with respect to introgression” (Hibbins and Hahn, 2022). However, likelihood-based methods such as bpp can accommodate multiple samples per species, and both theoretical analysis and computer simulation suggest that including multiple samples per species (in particular for species receiving immigrants) may boost the information content in the data for inferring gene flow (Huang *et al.*, 2020; Yang and Flouri, 2022). For example, with one sequence per species, some models of introgression are unidentifiable but the problem disappears when multiple samples are included in the data (Yang and Flouri, 2022; Thawornwattana *et al.*, 2023). It is unclear whether the extreme sensitivity in the inference of the *D. lowei*  →  *D. affinis* (p→q) introgression to taxon sampling (Table S7) is due to the joint effects of the use of one sample per species and the "pseudohaploidization" of the haploid consensus sequences, as the "unusualness" of the chimeric sequences from the ingroup species may depend on inclusion or exclusion of sequences from more distant species. Note that haploid consensus sequences may be chimeric sequences that do not exist in natural populations and may thus appear highly unusual. They may show up on gene trees as long branches or deeply divergent lineages, and may thus affect inference methods such as bpp that are based on gene genealogies (Huang *et al.*, 2022b, Fig. 6, Table 6).

While issues related to data quality may impact our analyses using bpp, the major introgression event involving sister lineages inferred in our analysis (Fig. 1b) appears to be robust and well supported. However, it is likely that certain instances of gene flow may be missed in our analyses. We leave it to future studies to assemble sequence datasets including noncoding parts of the genome and including multiple samples per species to infer gene flow in this group of species. In this regard, we note that (Kim *et al.*, 2024) has discussed the complexities of *Drosophila* genome assembly and made progress in producing high-quality genomic data.

## Supplementary Material and Data availability

Supplemental information is available at https://doi.org/10.5281/zenodo.13992534. Supplementary data (sequence alignments for the two data halves for clade 2 and bpp control files) are available through the Dryad Digital Repository at https://datadryad.org/dataset/doi:10.5061/dryad.ngf1vhj33.

## Funding

This study has been supported by the Biotechnology and Biological Sciences Research Council (BBSRC) grants (BB/T003502/1, BB/X007553/1, BB/R01356X/1) and a Natural Environment Research Council (NERC) grant (NE/X002071/1) to Z.Y.
